# Metabolic reprogramming and lung cancer focused on roles, mechanism, and clinical prospects of circRNAs: a narrative review

**DOI:** 10.3389/fonc.2026.1737600

**Published:** 2026-01-23

**Authors:** Simin Chen, Mingxiao Li, Siyao Li, Yinhui Sun, Lihuai Wang

**Affiliations:** 1 Medical School, Hunan University of Chinese Medicine, Changsha, China; 2 Oncology Medical Center, The First Hospital of Hunan University of Chinese Medicine, Changsha, China

**Keywords:** circRNA, lung cancer, mechanism, metabolic reprogramming, role

## Abstract

Lung cancer remains one of the malignancies with the highest incidence and mortality rates worldwide, and its treatment continues to pose significant challenges. Metabolic reprogramming, as one of the hallmarks of cancer, supports the abnormal growth, proliferation, invasion, and drug resistance of cancer cells by altering glucose, lipid, and amino acid metabolic pathways, providing both energy and biosynthetic precursors. It has thus become a critical focus in lung cancer research. Circular RNAs (CircRNAs), owing to their unique closed-loop structure and high stability, play important roles in regulating tumor metabolism and progression. This review systematically summarizes the molecular mechanisms through which CircRNAs drive metabolic reprogramming in lung cancer, including the regulation of key metabolic enzymes, influence on metabolism-related signaling pathways, remodeling of the tumor microenvironment, and mediation of epigenetic modifications. Furthermore, CircRNAs demonstrate great potential in clinical applications for lung cancer, not only as biomarkers for early diagnosis and prognostic evaluation but also as promising therapeutic targets. Leveraging their stability and low immunogenicity, the development of CircRNA-based vaccines and targeted delivery systems has opened new avenues for lung cancer immunotherapy. However, challenges remain in the synthesis of CircRNAs, understanding their *in vivo* metabolism, and achieving multi-target synergistic interventions, which warrant further investigation. This review provides a theoretical foundation for in-depth exploration of the metabolic regulatory network in lung cancer and the development of precise therapeutic strategies, while also highlighting the broad prospects of CircRNAs in translational medicine. We conducted a literature search across databases including PubMed up to 2025, focusing on keywords related to circRNA, lung cancer, and metabolic reprogramming. Ultimately, 161 relevant references were included in this narrative review.

## 1 Introduction

According to global cancer epidemiological reports, lung cancer ranks among the most common malignant tumors worldwide, with an incidence rate of 12.36% and a mortality rate as high as 17.59%, consistently representing the leading cause of cancer-related disease burden and constituting a major global health challenge (1–3). Although treatment strategies for lung cancer include surgery, chemotherapy, targeted therapy, and other modalities, the prognosis remains unsatisfactory, with a five-year survival rate of only approximately 20% (4, 5). Current therapeutic approaches face multiple challenges, such as strict limitations on surgical indications, significant side effects associated with chemotherapy, and the tendency of drug treatments to induce resistance. These factors severely constrain treatment efficacy and survival duration in lung cancer patients. Therefore, it is crucial to investigate the mechanisms underlying lung cancer development and progression and to identify novel biomarkers and therapeutic targets. Metabolic reprogramming, by modulating cellular energy metabolism, maintaining redox homeostasis, and regulating intracellular signal transduction, promotes rapid cancer cell growth and proliferation, making it one of the hallmarks of cancer (6, 7). Compared to normal cells, lung cancer cells—including adenocarcinoma, squamous cell carcinoma, and small cell lung cancer—exhibit significant alterations in metabolic patterns, characterized by enhanced glycolysis, upregulated amino acid metabolism, and dysregulated lipid metabolism (8). Notably, the regulation of oxidative phosphorylation (OXPHOS) in lung cancer demonstrates high heterogeneity: in certain subtypes, such as adenocarcinoma harboring mutations in epidermal growth factor receptor (EGFR) or Kirsten rat sarcoma viral oncogene homolog (KRAS). OXPHOS activity is not suppressed but instead compensatorily enhanced through increased mitochondrial biogenesis or upregulation of electron transport chain complexes, thereby sustaining tumor stemness and metastatic potential. In other subtypes, however, OXPHOS function is impaired due to microenvironmental hypoxia or suppression by oncogenic signals. This dynamic reprogramming further drives lung cancer progression by meeting the demands for biosynthetic precursors, energy, and redox capacity required for rapid proliferation, invasion, and metastasis (9).

Building upon the aberrant pathways associated with metabolic reprogramming in lung cancer, targeted therapies addressing these dysregulations have become a major research focus. Benefiting from their unique closed-loop structure, CircRNAs are resistant to degradation by Ribonuclease R (RNase R), enabling their stable presence in various tissues (10) and demonstrating relative stability in extracellular environments (11). In recent years, the role of CircRNAs in cancer research has gained increasing prominence. Studies have shown that CircRNAs play important roles in regulating tumor gene expression, including modulating tumor cell proliferation, invasion, migration, and apoptosis (12, 13). Furthermore, CircRNAs participate in regulating metabolic reprogramming in various cancer cells by altering energy acquisition and utilization mechanisms, thereby influencing the progression of malignant tumors (14–17). For instance, in lung adenocarcinoma, CircRNA_103809 functions as a competing endogenous RNA (ceRNA) by sponging miR-377-3p, thereby relieving its transcriptional repression of hexokinase and lactate dehydrogenase, ultimately driving the glycolytic process (18). Simultaneously, CircRNA_104135 forms a complex with the RNA-binding protein Fused in Sarcoma (FUS) to directly enhance the stability of glutaminase mRNA, consequently activating glutaminolytic metabolism (14). Research in lung squamous cell carcinoma indicates that CircRNA_0007534 upregulates pyruvate dehydrogenase kinase via miR-6855-3p-dependent epigenetic regulation, leading to impaired mitochondrial OXPHOS and a reinforced glycolytic phenotype. Additionally, CircCD36, through its sponge effect on miR-195-5p, significantly elevates the expression level of the lipid transporter CD36, promoting exogenous fatty acid uptake to meet tumor lipid metabolic demands (19). Further analysis in small cell lung cancer reveals that CircRNA_0072088 targets and inhibits miR-338-3p, thereby alleviating its negative regulation of enolase, which accelerates glycolytic flux and sustains rapid tumor proliferation (20). These studies unveil the intricate relationship between CircRNAs and lung cancer metabolic reprogramming. However, the specific mechanisms of CircRNAs in lung cancer metabolic reprogramming have not been fully elucidated, and their potential applications in clinical diagnosis, treatment, and prognosis evaluation warrant further exploration.

This review aims to systematically explore the roles, mechanisms, and potential clinical applications of Circular RNAs (CircRNAs) in metabolic reprogramming and lung cancer progression. By analyzing the functions and mechanisms of CircRNAs in regulating key metabolic enzymes, modulating metabolism-related signaling pathways, remodeling the tumor microenvironment, and mediating epigenetic modifications, we delve into their potential for clinical applications in lung cancer. This study will provide a novel theoretical foundation and research perspectives for precision medicine in lung cancer, with the goal of developing more effective diagnostic and therapeutic strategies.

In this review, we summarized recent literature retrieved from databases such as PubMed up to 2025. The search strategy combined keywords related to circular RNAs (e.g., “circRNA”, “circular RNA”), lung cancer subtypes (e.g., “NSCLC”, “lung adenocarcinoma”, “SCLC”), and key metabolic processes. Specifically, we used search terms such as “metabolic reprogramming”, “glucose metabolism”, “lipid metabolism”, and “Amino acid metabolism” to capture the diverse metabolic roles of circRNAs. We prioritized the inclusion of high-impact original research and authoritative reviews published within the past five years, with a specific focus on English-language literature that elucidates the mechanisms, functions, and clinical prospects of circRNAs in the metabolic reprogramming of lung cancer. Ultimately, 161 relevant references were included in this narrative review.

## 2 Overview of circular RNA

CircRNAs are a special class of non-coding RNA molecules. Unlike traditional linear RNAs, CircRNAs are formed through back-splicing, resulting in a closed circular structure that lacks a 5′-cap and a 3′-polyadenylated tail (21). This unique structure confers high stability to CircRNAs, making them resistant to degradation by exonucleases and allowing them to persist in cells for extended periods (22).

**Figure 1:**
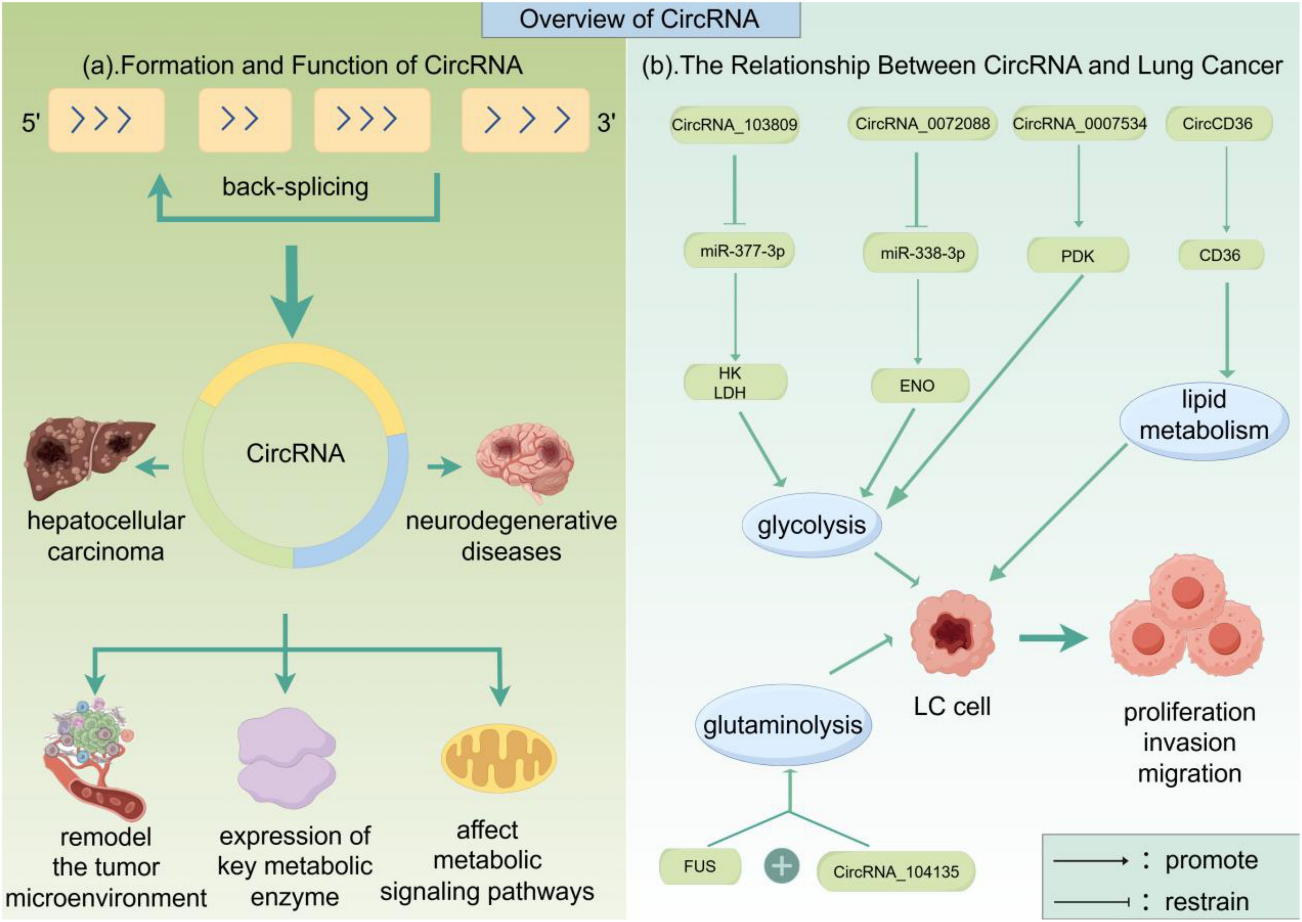
Overview of CircRNA. This figure summarizes the biogenesis, functions, and role in lung cancer of CircRNAs. They form stable closed loops through back-splicing of pre-mRNA. CircRNAs function as ceRNAs to sequester miRNAs and regulate gene expression; they also bind RNA-binding proteins to modulate gene function; some serve as templates for protein/peptide translation; a subset can directly participate in transcriptional regulation. Through these mechanisms, circRNAs broadly regulate processes such as proliferation, metabolism, and metastasis in lung cancer. Image was created with Figdraw.

Functionally, CircRNAs play key regulatory roles in various pathophysiological processes and exhibit diverse biological significance. Numerous studies have shown that (23, 24)CircRNAs not only act as miRNA sponges—dynamically participating in cholesterol synthesis, lipid metabolism, and inflammatory responses in atherosclerosis, thereby serving as critical regulatory nodes—but also play important roles in the following diseases: In cancer, specific CircRNAs drive progression through dual mechanisms. For example, CircHIPK3 sponges oncogenic miR-124 to relieve phosphatase and tensin homolog (PTEN) silencing, promoting hepatocellular carcinoma proliferation (25), while CircPVT1 promotes estrogen receptor-positive breast tumorigenesis and drug resistance by targeting estrogen receptor 1 (ESR1) and mitochondrial antiviral signaling protein (MAVS), facilitating cancer progression via ceRNA and protein scaffolding mechanisms (26); In neurodegenerative diseases, aberrantly expressed CircRNAs regulate Tau phosphorylation (e.g., in Alzheimer’s disease) or influence α-synuclein aggregation (e.g., in Parkinson’s disease), thereby disrupting neuronal survival and synaptic function (27, 28). In metabolic disorders, CircRNAs participate in maintaining glucose homeostasis by regulating insulin secretion-related genes (e.g., INSR) or glucose transporter expression (e.g., GLUT4) (29); In the cardiovascular system, CircRNAs modulate the TGF-β/Smad signaling axis to influence myocardial fibrosis and vascular remodeling processes (30). These multidimensional regulatory mechanisms highlight the broad and significant biological roles of CircRNAs as biomarkers or therapeutic targets within complex disease networks. miRNAs are a class of small non-coding RNAs that regulate gene expression through post-transcriptional mechanisms. Their core function relies on sequence-specific binding to the 3′-untranslated region (3′-UTR) of target mRNAs, leading to translational repression or mRNA degradation, thereby achieving gene silencing (31). During tumorigenesis and cancer progression, miRNAs exhibit a “molecular double-edged sword” characteristic: tumor-suppressive miRNAs (e.g., the let-7 family) effectively inhibit tumor cell proliferation and metastasis by silencing oncogenic networks such as K-RAS and c-Myc (32, 33), while oncomiRs (e.g., miR-21) drive malignancy progression in cancers such as breast and liver cancer by repressing tumor suppressors including PTEN and TPM1 (34–36). Mechanistic studies further reveal that miR-124 significantly inhibits the invasive ability of liver cancer cells by negatively regulating the epigenetic modifier EZH2, and miR-34a induces G_1_ phase arrest and activates p53-dependent apoptosis by directly binding to the 3′-UTRs of cell cycle regulators such as MYC and CDC25A (37–39). These findings systematically illustrate the central role of miRNAs in constructing multi-layer regulatory networks involved in tumor initiation, differentiation, and remodeling of the metastatic microenvironment (40). Notably, miRNA expression profiles are closely associated with molecular subtypes of tumors, treatment sensitivity, and prognosis evaluation, providing a theoretical basis for the development of liquid biopsy biomarkers and RNA interference-based therapies (41). The canonical function of circRNAs is acting as competing endogenous RNAs (ceRNAs). By specifically binding to microRNAs (miRNAs) and inhibiting their regulatory effects on target genes, circRNAs indirectly modulate gene expression (42). Additionally, circRNAs can directly interact with circRNA-binding proteins (cRBPs) to regulate gene functions (43). Recent studies have demonstrated that certain circRNAs containing internal ribosome entry sites (IRES) or N6-methyladenosine (m6A) modifications can serve as templates for translation, thereby encoding functional proteins or peptides. Furthermore, some circRNAs participate directly in transcriptional regulation (43). For instance, the nuclear-localized circFGFR1 inhibits the transcription of its host gene by forming an RNA-DNA triplex structure (R-loop) within the promoter region (44). (as shown in **Figure 1**).

With the continuous advancement of high-throughput sequencing technologies and bioinformatic analytical methods, an increasing number of CircRNAs have been identified and characterized (45). These CircRNAs exhibit unique biological functions in the development and progression of diseases. In the context of cancer, Long et al. (46)revealed that circ_0007379 acts as a scaffold to facilitate the processing of pri-miR-320a and pre-miR-320a in a KSRP-dependent manner, leading to enhanced maturation of miR−320a, which subsequently suppresses the expression of the transcription factor RUNX1, thereby inhibiting colorectal cancer progression. Fang et al. (47)demonstrated that EIF4E-mediated biogenesis of circPHF14 promotes the growth and metastasis of pancreatic ductal adenocarcinoma through the Wnt/β-catenin signaling pathway. Additionally, Chen et al. (48) found that circZNF707 facilitates lung cancer progression by sponging miR-668-3p, resulting in the upregulation of PFKM.

## 3 Metabolic reprogramming and lung cancer progression

Lung cancer is one of the most common malignant tumors worldwide, with high incidence and mortality rates (49). It is primarily classified into two types: small cell lung cancer (SCLC) and non-small cell lung cancer (NSCLC). NSCLC is the most prevalent subtype, accounting for 85–90% of all lung cancer cases. NSCLC consists of several histological subtypes, including lung adenocarcinoma (LUAD), lung squamous cell carcinoma (LSCC), and large cell lung cancer (LCLC) (50).

Tumor metabolic reprogramming refers to the adaptation of metabolic processes by cancer cells to meet the high energy demands of rapid proliferation. Research in this field dates back to the 1920s, when German biochemist Otto Warburg first described the tendency of tumor cells to generate energy via glycolysis even under oxygen-replete conditions—a phenomenon termed the “Warburg effect” (51–53). However, subsequent studies have revealed that (54–56) the Warburg effect and OXPHOS can be simultaneously upregulated in certain tumor cells. While relying on glycolysis for rapid energy supply, these cells retain and enhance the OXPHOS pathway. Consequently, OXPHOS generates substantial adenosine triphosphate (ATP) to fuel energy-intensive processes such as tumor invasion and metastasis.

Metabolic plasticity enables tumor cells to tailor their bioenergetics to specific stages of progression. In the invasive phase, cells rely heavily on fatty acid oxidation (FAO). Upregulated transporters increase fatty acid uptake for β-oxidation and tricarboxylic acid (TCA) cycle entry, generating ATP via mitochondrial respiration to power migration. Furthermore, FAO intermediates contribute to membrane remodeling, optimizing adhesion and detachment dynamics (57). In contrast, during metastatic colonization—particularly in the oxygen-rich environment of the lung—tumor cells shift toward glutamine metabolism. Through high transporter expression, glutamine is converted to α-ketoglutarate (α-KG) that fuels the TCA cycle. This process supports both energy generation and macromolecule biosynthesis, while the byproduct ammonia helps maintain intracellular pH homeostasis in the new environment (58).

**Figure 2:**
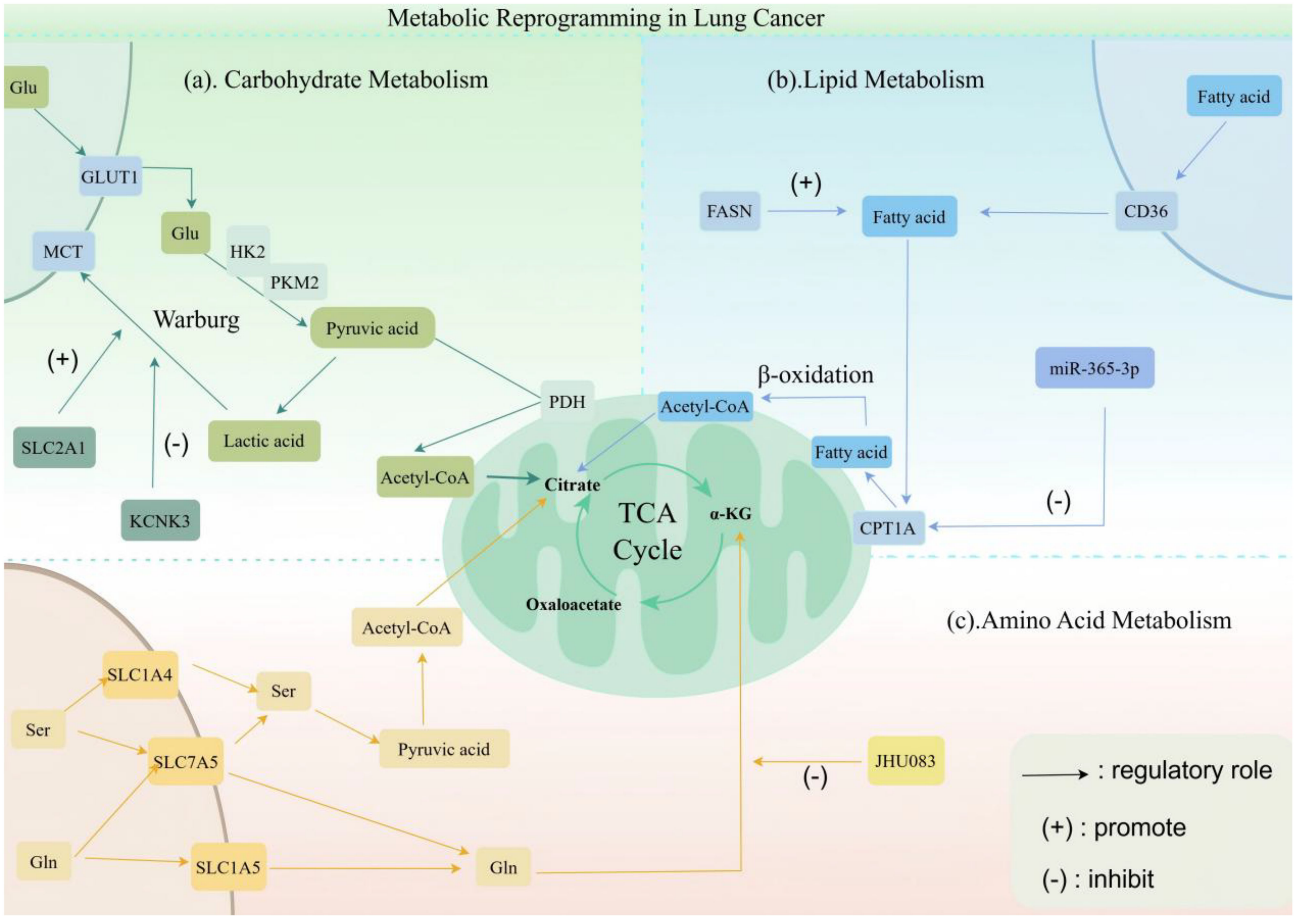
Metabolic reprogramming in lung cancer. This figure describes the hallmarks of metabolic reprogramming in lung cancer cells. Key features include enhanced aerobic glycolysis for rapid energy production (the Warburg effect) even under oxygen-sufficient conditions, alongside altered lipid and amino acid metabolism to meet biosynthetic demands. These interconnected metabolic alterations collectively drive malignant progression and reshape the tumor microenvironment. Image was created with Figdraw.

In summary, tumor metabolic reprogramming is characterized by significant spatiotemporal heterogeneity and context dependency. Tumor cells do not rely on a static metabolic profile; instead, they adaptively tailor their metabolic networks to meet distinct microenvironmental conditions and functional requirements. This plasticity involves the co-activation of glycolysis and OXPHOS, as well as the selective engagement of fatty acid oxidation or glutamine metabolism at specific stages, providing the energy and substrates necessary for survival and spread. Thus, metabolic reprogramming serves as a fundamental hallmark that underpins lung cancer initiation, progression, metastasis, and therapeutic resistance (59, 60).(as shown in **Figure 2**).

### 3.1 Glucose metabolism and lung cancer

Glucose metabolism is a fundamental physiological process essential for sustaining life, comprising both catabolism and anabolism. In catabolism, glycolysis converts glucose into pyruvate within the cytoplasm. Under anaerobic conditions, pyruvate is reduced to lactate, whereas under aerobic conditions, it enters the mitochondria and converts to acetyl-CoA to fuel the TCA cycle—the common pathway for complete substrate oxidation and substantial ATP generation (61). Regarding anabolism, glycogenesis polymerizes glucose to store energy via enzymatic reactions (62), while glycogenolysis functions in reverse to rapidly release glucose for blood glucose homeostasis (63). Additionally, gluconeogenesis synthesizes glucose from non-carbohydrate precursors to ensure energy supply during specific physiological states (64). These interconnected pathways are precisely regulated through mechanisms such as allosteric modulation, covalent modification, and hormonal control of key enzymes. This coordination adapts glucose metabolism to the organism’s varying energy and metabolic demands, ensuring normal physiological function.

Reprogramming of glucose metabolism is a hallmark of lung cancer. Lung cancer cells exhibit the “Warburg effect”, prioritizing glycolysis even in the presence of oxygen (65). Enhanced glycolytic flux enables rapid glucose uptake and its conversion to lactate, accompanied by limited ATP production (66). Beyond fueling rapid tumor proliferation, this process supplies abundant metabolic intermediates for biosynthesis. For instance, the pentose phosphate pathway derives ribose for nucleic acid synthesis, while glycolytic intermediates serve as precursors for amino acid and fatty acid synthesis (67). In the context of non-small cell lung cancer (NSCLC), Wang et al. (68) demonstrated via immunohistochemistry that protein tyrosine phosphatase receptor type H (PTPRH) upregulates glycolysis-related proteins (GLUT1, HK2, PKM2, and LDHA), thereby promoting tumor proliferation, migration, and invasion. Lung adenocarcinoma (LUAD), a common subtype of NSCLC, has been shown to be influenced by potassium channel activity. Lin et al. (69)reported that the acid-sensitive potassium channel 1 (KCNK3) suppresses cancer cell proliferation and glucose metabolism by activating the AMPK-TXNIP pathway in LUAD cells. Furthermore, Wang et al. (70) revealed that knocking down SLC2A1 expression in LUAD cells significantly impairs glucose transport, consumption, and lactate secretion, highlighting its pivotal role in glycolysis. Collectively, these findings suggest that targeting shared regulatory nodes linking glucose metabolism to lung cancer pathology—particularly in NSCLC and LUAD—may offer a promising strategy for modulating glucose metabolism and improving therapeutic outcomes.

### 3.2 Lipid metabolism and lung cancer

Lipid metabolism encompasses the digestion, absorption, synthesis, and breakdown of lipids—including fats, phospholipids, sphingolipids, and cholesteryl esters—in living organisms (71). Triglycerides are primarily synthesized in the liver via the monoacylglycerol and diacylglycerol pathways using products derived from glucose metabolism. Fatty acid synthesis occurs in the cytoplasm, starting from acetyl-CoA as the substrate (72). In catabolic pathways, triglyceride mobilization is catalyzed by hormone-sensitive lipase, while fatty acids are oxidized through β-oxidation and other pathways to generate energy. Unsaturated fatty acids undergo specific oxidative processes, and under certain conditions, the liver produces ketone bodies to supply extrahepatic tissues (73). These pathways work in concert to maintain lipid homeostasis, and dysregulation at any step may lead to various health disorders.

In addition to glucose reprogramming, lung cancer cells undergo profound changes in lipid metabolism to support membrane biogenesis, signaling, and energy storage (74). This is achieved by upregulating fatty acid uptake transporters and activating *de novo* synthesis pathways (75). Furthermore, tumor cells flexibly adjust lipid enzyme activity and metabolic flux to facilitate growth and dissemination. For instance, Xu et al. (76)demonstrated that miR-365-3p suppresses CPT1A expression by targeting its 3′-untranslated region in lung cancer cells, leading to increased lipid droplet accumulation, reduced ATP production, and decreased fatty acid oxidation, ultimately regulating cell proliferation and migration. Similarly, Wang et al. (77)identified CCAAT enhancer-binding protein δ (C/EBPδ) as a key lipid regulator. By recruiting nuclear receptor coactivator 3 (NCOA3), C/EBPδ transcriptionally activates Slug (a classic EMT transcription factor), which induces the expression of oxidized low-density lipoprotein (oxLDL) receptor-1 (Lox1) and enhances oxLDL uptake to promote metastasis. Additionally, using multi-omics approaches and lung epithelial-specific Cpt1a-knockout mouse models, Ma et al. (78)confirmed that CPT1A, the rate-limiting enzyme of FAO, collaborates with L-carnitine derived from tumor-associated macrophages to drive ferroptosis resistance and CD8^+^T cell inactivation in lung cancer. Therefore, elucidating the mechanisms linking lipid metabolism to lung cancer may provide a theoretical basis for developing innovative therapeutic strategies targeting key lipid metabolic regulators.

### 3.3 Amino acid metabolism and lung cancer

Amino acid metabolism is a critical physiological process encompassing both anabolism and catabolism. In terms of anabolism, non-essential amino acids are synthesized endogenously via pathways such as transamination and reductive amination, whereas essential amino acids must be acquired through dietary intake. During catabolism, amino acids initially undergo deamination to yield ammonia and corresponding α-keto acids. Ammonia is primarily converted into urea in the liver via the urea cycle (ornithine cycle) and excreted to maintain nitrogen balance. Meanwhile, the resulting α-keto acids serve as substrates for gluconeogenesis, ketogenesis, or OXPHOS supplying energy and biosynthetic raw materials (79). Collectively, this precisely regulated system is essential for sustaining vital life activities, regulating physiological functions and systemic homeostasis.

Amino acid metabolism also plays a pivotal role in lung cancer progression, with tumor cells exhibiting an increased demand for specific amino acids. Using high-performance liquid chromatography-mass spectrometry (HPLC-MS), Sun et al. (80)analyzed 23 amino acids in bronchoalveolar lavage fluid from lung cancer patients. Their results, validated through partial least squares-discriminant analysis (PLS-DA), Shapiro-Wilk tests, and Bonferroni correction, revealed significantly elevated serine levels in the lung cancer group. Serine serves not only as a nitrogen source for multiple biosynthetic pathways but also enters the TCA cycle to support energy production (81). Lung cancer cells enhance serine uptake by upregulating serine transporter expression. Concurrently, key enzymes involved in serine metabolism undergo adaptive changes, enabling efficient utilization of serine in support of tumor metabolic reprogramming (82).

Furthermore, glutamine plays a central role in the metabolic reprogramming of lung cancer cells. As the most abundant free amino acid in cells, glutamine serves not only as a critical substrate for protein and nucleotide synthesis but also as a key regulator of intracellular redox homeostasis (83, 84). Huang et al. (85)demonstrated that glutamine blockade using 6-diazo-5-oxo-L-norleucine (JHU083) significantly potentiates the efficacy of an EGFR peptide vaccine (EVax) in controlling EGFR-driven lung cancer. This blockade enhances immunoprevention by promoting the infiltration of anti-tumor CD8+T cells and Th1 cells while reducing immunosuppressive cell populations. Additionally, Liu et al. (86)revealed that cancer-associated fibroblast (CAF)-specific long non-coding RNA LINC01614, packaged in CAF-derived exosomes, directly interacts with ANXA2 and p65 to promote NF-κB activation. This leads to upregulation of the glutamine transporters SLC38A2 and SLC7A5, enhances glutamine uptake in cancer cells, and ultimately contributes to an unfavorable prognosis in LUAD.

In summary, amino acid metabolism plays a crucial role in lung cancer progression. Further elucidation of the regulatory mechanisms linking additional amino metabolic pathways to lung cancer may open up novel and effective therapeutic avenues for precision treatment of this malignancy.

## 4 CircRNA and metabolic reprogramming in lung cancer

**Figure 3:**
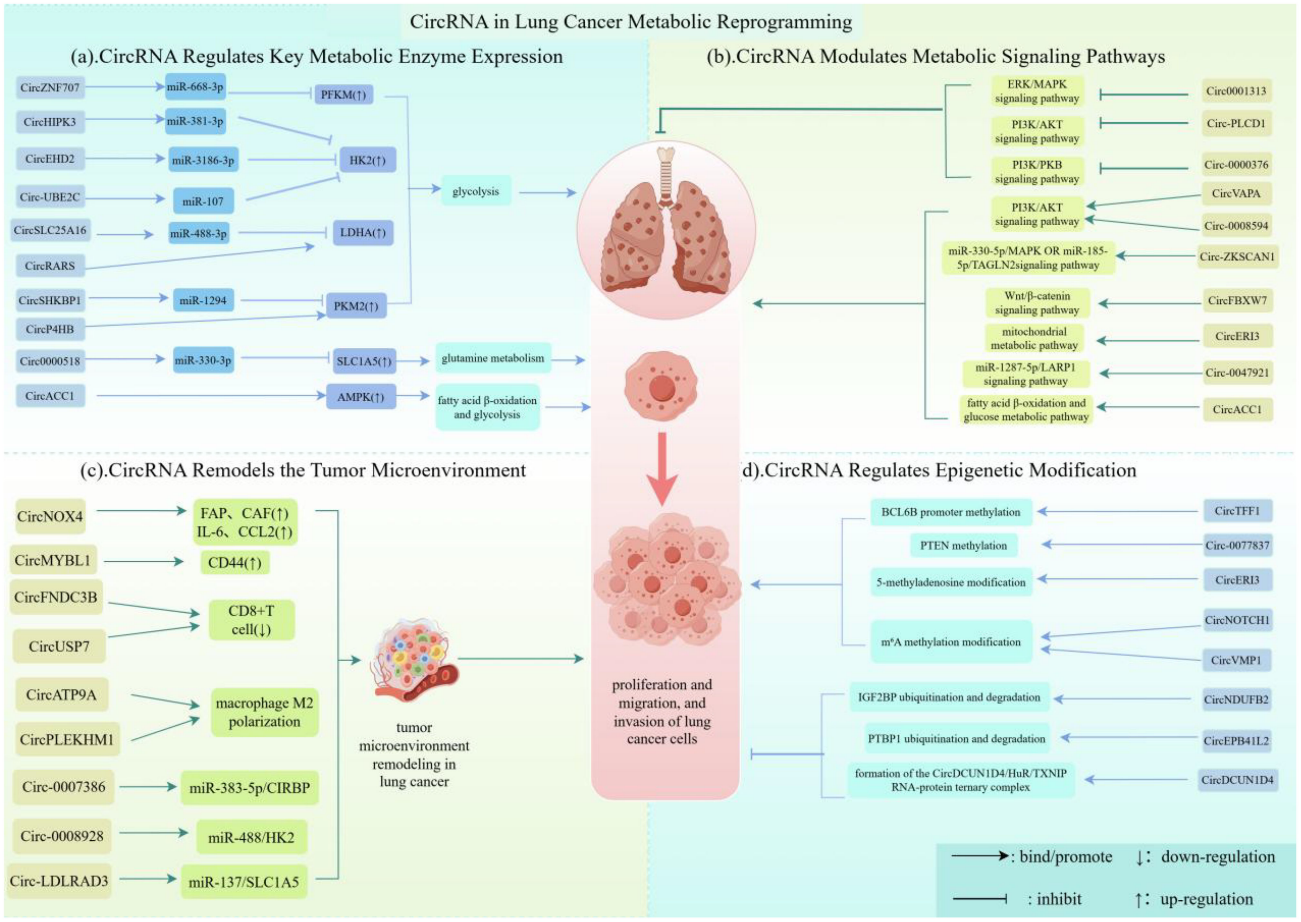
CircRNA in lung cancer metabolic reprogramming. This figure illustrates the multi-layered regulatory network through which CircRNAs drive metabolic reprogramming in lung cancer. CircRNAs coordinately alter the metabolic state of cancer cells by regulating key metabolic enzymes, modulating related signaling pathways, remodeling the tumor microenvironment, and mediating epigenetic modifications, thereby promoting tumor proliferation and adaptation. Image was created with Figdraw.

In recent years, CircRNAs, as rising stars in the non-coding RNA family, have been demonstrated to play indispensable regulatory roles in the progression of numerous diseases—from autoimmune and cardiovascular diseases to osteoarticular disorders (87)—and particularly in cancer (88). Through summarizing and analyzing current literature on the mechanisms of CircRNAs in metabolic reprogramming in lung cancer, we aim to elucidate their diverse modes of action and impacts on lung cancer progression, thereby exploring the enigmatic functions of CircRNAs in the metabolic landscape of lung cancer. Differentially expressed CircRNAs in NSCLC and other lung cancer cells mediate biological effects ranging from modulating the expression levels of key intracellular enzymes and metabolically relevant signaling pathways to remodeling the metabolic tumor microenvironment. This extensive regulatory network, spanning both intracellular and extracellular processes, compellingly directs our attention to these molecular messengers in this microscopic world (89, 90).(as shown in **Figure 3**).

### 4.1 CircRNA regulates the expression of key enzymes in metabolism

In lung cancer cells, circRNAs such as CircZNF707 and CircHIPK3 play a crucial role in regulating the expression of key metabolic enzymes. Mostly by altering the expression levels and activity of metabolic enzymes such as PFKM and HK2, they can meet the energy and material demands for rapid proliferation, invasion, and metastasis of lung cancer cells (91). But how exactly do circRNAs exert their regulatory functions in lung cancer cells? Further studies have revealed that circRNAs can participate in the regulation of key metabolic enzyme expression through multiple mechanisms. On one hand, circRNAs can act as molecular sponges to adsorb microRNAs (miRNAs), thereby alleviating the inhibitory effect of miRNAs on the mRNA of key metabolic enzymes and promoting their expression. For example, CircZNF707 competitively binds to miR-668-3p, upregulates PFKM expression, promotes glycolysis, and enhances the proliferation, migration, and invasion of non-small cell lung cancer cells (48); Researchers such as Gu et al. found that CircHIPK3, by adsorbing miR-381-3p, relieves the negative regulation of HK2 by miR-381-3p, upregulates HK2 expression, modulates glycolytic metabolism, and promotes lung cancer cell proliferation and migration (92); Similarly, CircSHKBP1 upregulates PKM2 expression by sponging miR-1294, mediating glycolysis and promoting the growth and metastasis of non-small cell lung cancer cells (93); Likewise, CircEHD2 adsorbs miR-3186-3p, upregulates HK2 expression, and facilitates glycolysis and the proliferation of non-small cell lung cancer cells (94); Moreover, Circ_UBE2C captures miR-107, alleviating its inhibition of HK2 and upregulating HK2 expression, thereby promoting glycolysis and lung cancer cell proliferation (95); It is noteworthy that some circRNAs exhibit more complex regulatory hierarchies. For instance, CircSLC25A16 can activate LDHA and promote its transcription through the miR-488-3p/HIF1α/LDHA signaling axis, enhancing glycolysis while significantly promoting the spread of non-small cell lung cancer (96); Meanwhile, Circ0000518 upregulates SLC1A5 expression by modulating the miR-330-3p/SLC1A5 axis, regulating glutamine metabolism and promoting the progression of non-small cell lung cancer (97). On the other hand, as a special class of non-coding RNAs, circRNAs can interact with specific proteins to regulate the expression of key metabolic enzymes. For example, CircP4HB binds to PKM2 and subsequently upregulates its expression by enhancing tetramer formation, promoting tumor progression in lung adenocarcinoma (98); Researchers such as Li et al. found that CircACC1 binds to the β and γ subunits of AMPK, stabilizing AMPK and enhancing its activity, promoting fatty acid β-oxidation and glucose metabolism while inhibiting lipid synthesis, thereby regulating metabolic reprogramming in lung cancer cells (99). Additionally, circRNAs can directly influence transcription and translation processes to regulate the expression of key metabolic enzymes. For instance, CircRARS positively regulates LDHA activity and expression at the transcriptional level, promoting glycolysis and the proliferation of non-small cell lung cancer cells (100). In summary, in lung cancer tissues, these circRNAs directly or indirectly regulate the expression of key metabolic enzymes through various molecular mechanisms, promoting metabolism such as glycolysis, which serves as the primary nutrient source for the proliferation and spread of lung cancer cells.(as shown in **Figure 3** and **Table 1**).

**Table 1:** Regulation of key metabolic enzymes by CircRNA.

CircRNA	Mechanism	Sponged miRNA/interacting protein	Regulated key metabolic enzyme	Functions	References
CircZNF707	As a molecular sponge, miRNA is adsorbed to relieve the inhibition of miRNA on key metabolic enzymes	miR-668-3p	PFKM	Promotes glycolysis; enhances proliferation, migration, and invasion of NSCLC cells	(48)
CircHIPK3		miR-381-3p	HK2	Modulates glycolytic metabolism; promotes proliferation and migration of lung cancer cells	(92)
CircSHKBP1		miR-1294	PKM2	Mediates glycolysis; promotes growth and metastasis of NSCLC cells	(93)
CircEHD2		miR-3186-3p	HK2	Promotes glycolysis and proliferation of NSCLC cells	(94)
Circ-UBE2C		miR-107	HK2	Promotes glycolysis and proliferation of lung cancer cells	(95)
CircSLC25A16		miR-488-3p	LDHA	Promotes glycolysis and dissemination of NSCLC cells	(96)
Circ0000518		miR-330-3p	SLC1A5	Regulates glutamine metabolism; promotes NSCLC progression	(97)
CircP4HB	Interacts with specific proteins to regulate the expression of metabolic enzymes	PKM2	PKM2	Promotes progression of lung adenocarcinoma	(98)
CircACC1		β and γ subunits of AMPK	AMPK	Promotes fatty acid β-oxidation and glucose metabolism; inhibits lipid synthesis; reprograms lung cancer cell metabolism	(99)
CircRARS	Directly regulates transcriptional processes to control metabolic enzyme expression	–	LDHA	Promotes glycolysis and proliferation of NSCLC cells	(100)

### 4.2 CircRNA affects metabolic signaling pathways

In addition to influencing the metabolic processes of lung cancer cells by regulating the expression of key metabolic enzymes, circRNAs can also mediate metabolism-related signaling pathways such as PI3K, MAPK, and Wnt/β-catenin to modulate various metabolic processes in lung cancer cells, thereby regulating metabolic reprogramming in lung cancer. The PI3K pathway plays a crucial role in the proliferation, apoptosis, invasion, metastasis, and immune regulation of lung cancer cells, making it one of the key signaling pathways involved in the development and targeted therapy of lung cancer (101). Researchers have found that CircVAPA can activate the PI3K/AKT signaling pathway by regulating the miR-377-3p/IGF1R axis and the miR-494-3p/IGF1R axis, thereby modulating small cell lung cancer (102). For instance, Circ_0000376 inhibits the activity of the PI3K/PKB signaling pathway by downregulating the levels of phosphorylated PI3K and PKB, thereby suppressing the progression of non-small cell lung cancer (103). Circ-PLCD1, on the other hand, can adsorb miR-375 and miR-1179 and increase PTEN expression, thereby inhibiting the PI3K/AKT signaling pathway and acting as a tumor suppressor in non-small cell lung cancer (104). Additionally, miR-760 overexpression attenuates the regulatory effects of Circ_0008594 on the functions of H23 and H460 cells as well as the PI3K/AKT pathway. Therefore, Circ_0008594 promotes the development of non-small cell lung cancer by regulating the miR-760-mediated PI3K/AKT pathway (105).

The MAPK signaling pathway connects extracellular signals to cellular functions such as development, proliferation, differentiation, migration, and apoptosis. Abnormalities in the MAPK pathway can lead to cancer development (106). Circ0001313, Circ-ZKSCAN1, and others can mediate the MAPK signaling pathway to regulate the proliferation and development of lung cancer. For example, Circ0001313 competitively binds with miR-452, upregulates HMGB3 levels, and attenuates the ERK/MAPK signaling pathway, thereby inhibiting the proliferation and invasion of non-small cell lung cancer cells (107). Meanwhile, some scholars have found that Circ-ZKSCAN1, by adsorbing miR-330-5p, upregulates FAM83A expression, thereby inhibiting the MAPK signaling transduction pathway and further promoting the progression of non-small cell lung cancer (108).

Aberrations in the Wnt/β-catenin signaling pathway are not only a critical factor in the development and progression of lung cancer but also regulate cancer stem cell properties, invasion, and metastasis (109). Certain circRNAs can also mediate the Wnt/β-catenin signaling pathway and other metabolism-related pathways to regulate lung cancer progression. For instance, CircFBXW7 can be translated into a short peptide, circFBXW7-185aa, which, after epigenetic modification, interacts with β-catenin, leading to its ubiquitination and degradation. This process mediates the Wnt/β-catenin signaling pathway and modulates the progression of lung adenocarcinoma (110). Furthermore, circRNAs such as CircERI3 and CircACC1 can participate in regulating other signaling pathways, including mitochondrial metabolism and glucose metabolism, thereby providing necessary nutrients and energy support for the growth and migration of lung cancer cells. Specifically, CircERI3 interacts with DDB1, modulates its ubiquitination process, and enhances its stability, thereby promoting peroxisome proliferation, influencing mitochondrial function and metabolism, and ultimately driving lung cancer proliferation (111). Circ_0047921 mediates the miR-1287-5p/LARP1 signaling pathway, enhances glucose metabolism, and significantly promotes the proliferation, migration, and invasion of lung cancer cells (112). Additionally, researchers such as Li et al. found that CircACC1 directly binds to AMPK subunits, stabilizing and enhancing AMPK activity, which coordinates fatty acid β-oxidation and glucose metabolism while promoting lung cancer proliferation (99). It is noteworthy that some circRNAs can mediate multiple metabolic pathways simultaneously, rather than just one. For example, Circ-ZKSCAN1 not only promotes non-small cell lung cancer proliferation by adsorbing miR-330-5p and mediating the MAPK signaling pathway but also facilitates lung adenocarcinoma proliferation by regulating the miR-185-5p/TAGLN2 axis (108, 113).(as shown in **Figure 3** and **Table 2**).

**Table 2:** Regulation of signaling pathways by CircRNAs in lung cancer metabolic processes.

CircRNA	Mediated signaling pathway	Functions	References
CircVAPA	PI3K/AKT signaling pathway	Regulates small cell lung cancer	(102)
Circ-0000376	PI3K/PKB signaling pathway	Suppresses the development and progression of NSCLC	(103)
Circ-PLCD1	PI3K/AKT signaling pathway	Inhibits NSCLC	(104)
Circ-0008594	PI3K/AKT signaling pathway	Promotes NSCLC development	(105)
Circ0001313	ERK/MAPK signaling pathway	Inhibits proliferation and invasion of NSCLC cells	(107)
CircFBXW7	Wnt/β-catenin signaling pathway	Regulates progression of lung adenocarcinoma	(110)
CircERI3	Mitochondrial metabolic pathway	Promotes lung cancer proliferation	(111)
Circ-0047921	miR-1287-5p/LARP1 signaling pathway	Promotes proliferation, migration, and invasion of lung cancer cells	(112)
CircACC1	Fatty acid β-oxidation and glucose metabolism pathways	Promotes lung cancer proliferation	(99)
Circ-ZKSCAN1	miR-330-5p/MAPK or miR-185-5p/TAGLN2 signaling pathway	Promotes proliferation of NSCLC or lung adenocarcinoma	(108, 113)

### 4.3 CircRNA reshapes the tumor microenvironment

The tumor microenvironment (TME) comprises cellular components such as fibroblasts, endothelial cells, and immune cells, as well as non-cellular components including the extracellular matrix (ECM) and cytokines (114). During the development and progression of lung cancer, significant changes occur in the TME of lung cancer tissues, including alterations in cellular components, remodeling of the ECM, changes in cytokines, and metabolic reprogramming, which collectively provide the driving force for the proliferation and migration of lung cancer cells (115). CircRNAs contribute substantially to the remodeling of the TME, thereby creating favorable conditions for the growth, invasion, and metastasis of lung cancer cells (116). For instance, CircNOX4 upregulates fibroblast activation protein (FAP) via the miR-329-5p/FAP axis, increasing the expression of cancer-associated fibroblasts (CAFs). This enhances the glycolytic capacity and lactate secretion of CAFs, providing energy and synthetic precursors for tumor cells, thereby promoting angiogenesis, inflammatory responses, and metastasis, ultimately influencing the growth and metastasis of NSCLC (117). Extracellular vesicle-carried CircMYBL1 can modulate CD44 expression in human pulmonary microvascular endothelial cells (HPMECs), promoting adhesion between cancer cells and endothelial cells and facilitating pulmonary metastasis of adenoid cystic carcinoma (118). Moreover, circRNAs can modulate the functions of key immune cells, such as T cells and macrophages, either directly or through exosome-mediated mechanisms, thereby fostering an immunosuppressive TME in lung cancer. On one hand, circRNAs suppress T cell activity and infiltration. For instance, Wei et al. (119) demonstrated that circFNDC3B binds to transcription factor II-I (TFII-I) to downregulate the chemokines CXCL10 and CXCL11, consequently restricting CD8+T cell infiltration in NSCLC tissues. Similarly, circUSP7 induces CD8+T cell dysfunction via the miR-934/SHP2 axis, leading to resistance against anti-PD-1 therapy (120). On the other hand, circRNAs are pivotal in regulating macrophage polarization. Studies have shown that circATP9A and exosomal circPLEKHM1 (under hypoxic conditions) can facilitate macrophage polarization toward the pro-tumorigenic M2 phenotype via extracellular vesicle-mediated delivery, thereby driving lung cancer progression and metastasis (121, 122). CircRNAs can also facilitate the invasion and metastasis of lung cancer cells by breaking through ECM constraints via regulation of ECM remodeling. For example, under hypoxic conditions, Circ_0007386 enhances its circularization through YAP1-EIF4A3 interaction, subsequently affecting ECM remodeling via the miR-383-5p/CIRBP axis (123). Meanwhile, CircRNAs play a significant role in modulating cytokines. CircNOX4, through the miR-329-5p/FAP axis, upregulates FAP and promotes the secretion of cytokines such as IL-6 and CCL2, thereby fostering a pro-metastatic inflammatory microenvironment and further promoting the growth and metastasis of NSCLC (117). Additionally, some CircRNAs can alter the tumor metabolic microenvironment of lung cancer by regulating metabolism. For instance, Circ_0008928 promotes glycolytic metabolism through the miR-488/HK2 axis, remodeling the glucose metabolic microenvironment and facilitating the growth and migration of NSCLC cells (124). Furthermore, researchers such as Xue et al. found that Circ-LDLRAD3 promotes glutamine transport and metabolism by regulating the miR-137/SLC1A5 axis, reshaping the amino acid metabolic microenvironment in lung cancer. This not only provides nitrogen sources and energy for lung cancer cells but also participates in amino acid synthesis, supporting the biosynthetic and energy metabolism required for lung cancer cell proliferation, thereby promoting lung cancer progression (125). In summary, CircRNAs remodel the tumor microenvironment through diverse pathways, involving both cellular and non-cellular components, creating favorable conditions for the proliferation and metastasis of lung cancer cells.(as shown in **Figure 3** and **Table 3**).

**Table 3:** Mechanisms of CircRNAs in remodeling the tumor microenvironment in lung cancer.

CircRNA	Mechanism	Functions	References
CircNOX4	Upregulates FAP via the miR-329-5p/FAP axis, increasing CAF expression and promotes the secretion of cytokines such as IL-6 and CCL2	Promotes angiogenesis, inflammation, and metastasis; shapes a pro-metastatic inflammatory microenvironment; facilitates NSCLC growth and metastasis	(117)
CircMYBL1	Modulates CD44 expression in vascular endothelial cells (HPMEC)	Promotes adhesion between cancer cells and endothelial cells, facilitating lung metastasis in adenoid cystic carcinoma	(118)
CircFNDC3B	Binds to transcription factor II-I (TFII-I) to downregulate CXCL10 and CXCL11	Inhibits CD8+T cell infiltration in NSCLC tissues	(119)
CircUSP7	Regulates the miR-934/SHP2 axis	Induces CD8+T cell dysfunction and leads to anti-PD-1 immunotherapy resistance	(120)
CircATP9A and CircPLEKHM1	Delivered via extracellular vesicles (e.g., exosomes)	Promotes macrophage polarization toward the M2 phenotype to drive tumor progression and metastasis	(121, 122)
Circ-0007386	Affects extracellular matrix remodeling via the miR-383-5p/CIRBP axis	Promotes invasion and metastasis of lung cancer cells by breaking through matrix restrictions	(123)
Circ-0008928	Remodels the glucose metabolic microenvironment via the miR-488/HK2 axis	Promotes NSCLC cell growth and migration	(124)
Circ-LDLRAD3	Modulates the miR-137/SLC1A5 axis to reshape the amino acid metabolic microenvironment in lung cancer	Promotes lung cancer progression	(125)

### 4.4 CircRNA regulates epigenetic modification

Epigenetic modifications, including DNA methylation, histone modifications, and non-coding RNA-mediated gene regulation, represent a reversible and heritable mode of influencing gene expression (126). In the metabolic reprogramming of lung cancer, the regulation of epigenetic modifications by circRNAs is of great significance, as they exert regulatory roles at multiple levels such as DNA methylation, histone modifications, chromatin remodeling, and RNA modifications. Lung cancer tissues exhibit significantly abnormal histone acetylation, with promoter hypermethylation commonly observed in early stages leading to the inactivation of tumor suppressor genes, while promoter hypomethylation or loss of methylation is more frequent in advanced stages (127). Some circRNAs can regulate metabolism through epigenetic modifications such as DNA methylation, histone modifications, and chromatin remodeling, thereby promoting the proliferation and invasion of lung cancer cells (127). For instance, CircTFF1 upregulates DNMT3A via the miR-29c-3p/DNMT3A axis, promoting BCL6B promoter methylation and suppressing its transcription. As BCL6B is a transcriptional repressor, its downregulation alleviates the transcriptional repression of various metabolism-related genes, thereby remodeling the metabolic network of lung cancer cells and providing energy and nutrients for their proliferation, migration, and invasion (128). In NSCLC, upregulation of Circ_0077837 reduces PTEN expression and increases PTEN gene methylation, thereby inhibiting apoptosis in lung cancer cells (129). CircRNAs can also regulate lung cancer metabolism through RNA modifications. For example, CircERI3 undergoes increased nuclear export via 5-methyladenosine modification, enhancing DDB1 stability and promoting PGC-1α transcription, thereby altering mitochondrial energy metabolism and facilitating lung cancer development (111). Similarly, CircNOTCH1 regulates the NOTCH1 pathway by modulating m6A methylation, indirectly influencing glycolysis and promoting NSCLC cell growth (130). Additionally, CircVMP1 mediates the miR-524-5p/METTL3 axis by regulating m6A modification, promoting the progression of NSCLC (131). Beyond DNA methylation and RNA modifications, circRNAs in lung cancer tissues also regulate histone modifications and chromatin remodeling. For example, CircNDUFB2 inhibits NSCLC progression by enhancing the ubiquitination and degradation of IGF2BP (132). Meanwhile, CircEPB41L2 can bind to the RRM1 domain of PTBP1 and the E3 ubiquitin ligase TRIP12, promoting polyubiquitination and degradation of PTBP1, thereby inhibiting glucose uptake and lactate production, and subsequently suppressing NSCLC progression and metastasis (133). Conversely, CircDCUN1D4 forms a CircDCUN1D4/HuR/TXNIP RNA-protein ternary complex, stabilizing TXNIP expression, which inhibits glucose uptake and glycolysis, as well as the metastasis of lung cancer cells (134). The regulation of epigenetic modifications by circRNAs in lung cancer tissues constitutes a complex and precise network involving DNA methylation, histone modifications, and other mechanisms. This network not only influences the metabolism of lung cancer cells but also remodels the tumor microenvironment, promoting their proliferation and invasion.(as shown in **Figure 3**).

## 5 Clinical application prospects of circRNA

The evolutionarily conserved circRNA family exhibits stable expression differences across various tissues, thereby mediating countless biological functions. Not only can they express proteins like lncRNAs, but their superior circular structure also provides stable properties for *in vivo* delivery and low immunogenicity (135). To date, the clinical translation of circRNAs primarily includes disease diagnosis, prognosis assessment (136), vaccine development (137, 138), and targeted drug discovery, among other aspects. Owing to their stable expression patterns, we can now detect circRNA types in blood, saliva, and tissues to inform critical clinical decisions such as early disease diagnosis and prognosis evaluation. This has been studied in various diseases, including ophthalmic diseases (139), lung cancer (140), breast cancer (141), colorectal cancer (142), stroke (143), heart failure (144), multiple sclerosis (145), and major depressive disorder (146).

Compared to mRNA vaccines, circRNA vaccines not only offer advantages such as high efficacy, low immunogenicity, high stability, ease of production, and durability but also enable stable protein expression *in vivo*, triggering a more robust adaptive immune response and higher antibody production. Researchers such as Laura et al. (138)explored the adjuvant activity of circRNA vaccines administered in mice, using CART delivery systems to enhance translational activity. They successfully induced T-cell responses in mice, paving new pathways for clinical cancer immunotherapy. Although the development of such RNA vaccines is still in its early stages and has not yet entered clinical trials, they hold broad therapeutic potential for further translation in areas such as viral infections, cancer treatment, metabolic diseases, and autoimmune disorders (137).

CircRNAs hold promise for applications such as protein replacement therapy, vaccine development, cancer immunotherapy, and gene editing. Their *in vivo* delivery systems mainly include lipid nanoparticles, exosomes, virus-like particles, and viral vectors (147). For example, exosomes are natural intercellular communication vehicles—small, biocompatible, and innate—making them highly suitable as delivery carriers (148). However, intravenous administration has an extremely short duration, and chemical/biological modifications can extend their half-life (148). Due to the challenges of loading circRNAs into exosomes owing to their unique circular structure, researchers such as Yu et al. (149) constructed the coding DNA of the target circRNA DYM into a lentiviral vector. This vector was used to transfect T cells, enabling accurate and efficient circularization and facilitating large-scale production. Although circRNAs hold immense potential for clinical translation, they still face challenges in synthesis, purification, and delivery systems. Their metabolic processes, efficacy, and distribution *in vivo* require further exploration (147).

## 6 Conclusion and outlook

In this review, we summarize the biological roles mediated by differentially expressed circRNAs in lung cancer cells. A growing body of research has highlighted the role of non-coding RNAs in remodeling the tumor microenvironment (150). Among them, circRNAs promote the progression of malignant phenotypes in lung cancer by regulating glycolysis. Recent studies have shown that circRNAs facilitate glycolysis through their sponge effect on miRNAs, thereby promoting the progression of malignant phenotypes in lung cancer (151). Furthermore, circRNAs also regulate the expression of related enzymes. For instance, CircSLC25A16 upregulates LDHA and promotes glycolysis by modulating key glycolytic enzymes, thereby facilitating the initiation and progression of lung cancer (151). The implementation of this mechanism demonstrates that non-coding RNAs have the potential to regulate the mRNA and protein expression of metabolic enzymes or indirectly interact with key factors that modulate the synthesis of metabolic enzymes (150).

CircRNAs can collaborate with other molecules to remodel the tumor microenvironment. For example, circRNAs adsorb miRNAs to regulate the expression of miRNA target genes, thereby participating in the modulation of malignant behaviors and immune escape in various tumor cells, including lung cancer (152). Specifically, circRNAs can influence the regulatory effects of miRNAs on their target genes by binding to miRNAs, making them potential biomarkers (153). To realize this potential, a clear translational pathway emerges, guiding future efforts from validation to application: advancing the clinical validation and standardized detection of key circRNA biomarkers will rely on large-scale prospective studies (136) and the establishment of robust, reproducible liquid biopsy protocols (154); overcoming *in vivo* delivery hurdles necessitates the development of novel targeted systems, such as engineered nanocarriers, to provide precise therapeutic tools (147); integrating circRNA profiles with multi-omics data to construct prognostic models will chart a new course for precision diagnosis and therapy in lung cancer (155); ultimately, through innovative clinical trials, strategies combining interventions targeting circRNA-mediated metabolic axes with existing treatments hold promise for breaking through therapeutic plateaus and offering new hope to patients (156). Existing research indicates that while circRNAs hold great potential in inhibiting the activity of key metabolic enzymes in metabolic pathways, it is necessary to simultaneously target multiple metabolic pathways or combine interventions in oncogenic signaling pathways to enhance therapeutic efficacy and avoid drug resistance (157). Moreover, studies on circRNAs in lung cancer provide new insights and potential biomarkers for early diagnosis, prognosis assessment, and treatment of lung cancer (153). Additionally, circRNAs show promise in cancer therapy, including but not limited to the use of RNA interference (RNAi) and CRISPR-Cas9 systems to target specific circRNAs, thereby inhibiting cancer cell proliferation and invasion (158). These advancements offer hope for optimizing clinical prediction and cancer treatment. However, current research on the regulation of cancer metabolic reprogramming by circRNAs also faces many challenges. In recent years, few studies have explored their clinical application as diagnostic cancer biomarkers, and the complexity of experimental methods and difficulties in detection remain limitations that need to be addressed in future research (159).

In summary, our review provides a theoretical foundation for in-depth analysis of the metabolic regulatory network in lung cancer and the development of precision treatment strategies. Research on circRNAs contributes to a better understanding of altered energy metabolism in cancer cells. With advances in biological research, circRNAs are expected to be applied in clinical settings for regulating immunometabolism (158) and multi-target synergistic interventions, thereby contributing to cancer therapy.
