# Supplementary material for: Metabolic reprogramming and lung cancer focused on roles, mechanism, and clinical prospects of circRNAs: a narrative review
Source: Front Oncol. 2026 Jan 23;16:1737600. doi: 10.3389/fonc.2026.1737600 (PMC12875922; doi:10.3389/fonc.2026.1737600)
Supplement: Supplementary file 1 [file Table1.docx]

**Abbreviations list**

CircRNAs —— Circular RNAs

LncRNA —— Long Non-Coding RNA

OXPHOS —— oxidative phosphorylation

ceRNA —— endogenous RNA

miRNA —— microRNA

SCLC —— small cell lung cancer

NSCLC —— non-small cell lung cancer

LUAD —— lung adenocarcinoma

LSCC —— lung squamous cell carcinoma

LCLC —— large cell lung cancer

FAO —— fatty acid oxidation

TCA —— tricarboxylic acid

α-KG —— α-ketoglutarate

PTPRH —— protein tyrosine phosphatase H receptor

NCOA3 —— oncogenic nuclear receptor coactivator 3

EMT —— epithelial-mesenchymal transition

oxLDL —— oxidized low-density lipoprotein

HPLC-MS —— high-performance liquid chromatography-mass spectrometry

PLS-DA —— partial least squares-discriminant analysis

EGFR —— epidermal growth factor receptor

CAF —— cancer-associated fibroblast

TME —— tumor microenvironment

ECM —— extracellular matrix

FAP —— fibroblast activation protein

CAFs —— cancer-associated fibroblasts

HPMECs —— human pulmonary microvascular endothelial cells
